# “It Made Me Feel like Things Are Starting to Change in Society:” A Qualitative Study to Foster Positive Patient Experiences during Phone-Based Social Needs Interventions

**DOI:** 10.3390/ijerph191912668

**Published:** 2022-10-03

**Authors:** Anna L. Steeves-Reece, Christina Nicolaidis, Dawn M. Richardson, Melissa Frangie, Katherin Gomez-Arboleda, Chrystal Barnes, Minnie Kang, Bruce Goldberg, Stephan R. Lindner, Melinda M. Davis

**Affiliations:** 1 School of Public Health, Portland State University—Oregon Health & Science University, Portland, OR 97201, USA; 2 Oregon Rural Practice-Based Research Network, School of Medicine, Oregon Health & Science University, Portland, OR 97201, USA; 3 School of Social Work, Portland State University, Portland, OR 97201, USA; 4 Division of General Internal Medicine and Geriatrics, School of Medicine, Oregon Health & Science University, Portland, OR 97239, USA; 5 Center for Health Systems Effectiveness, Department of Emergency Medicine, School of Medicine, Oregon Health & Science University, Portland, OR 97239, USA; 6 Department of Family Medicine, School of Medicine, Oregon Health & Science University, Portland, OR 97239, USA

**Keywords:** health-related social needs, patient-centered care, qualitative research

## Abstract

Many healthcare organizations are screening patients for health-related social needs (HRSN) to improve healthcare quality and outcomes. Due to both the COVID-19 pandemic and limited time during clinical visits, much of this screening is now happening by phone. To promote healing and avoid harm, it is vital to understand patient experiences and recommendations regarding these activities. We conducted a pragmatic qualitative study with patients who had participated in a HRSN intervention. We applied maximum variation sampling, completed recruitment and interviews by phone, and carried out an inductive reflexive thematic analysis. From August to November 2021 we interviewed 34 patients, developed 6 themes, and used these themes to create a framework for generating positive patient experiences during phone-based HRSN interventions. First, we found patients were likely to have initial skepticism or reservations about the intervention. Second, we identified 4 positive intervention components regarding patient experience: transparency and respect for patient autonomy; kind demeanor; genuine intention to help; and attentiveness and responsiveness to patients’ situations. Finally, we found patients could be left with feelings of appreciation or hope, regardless of whether they connected with HRSN resources. Healthcare organizations can incorporate our framework into trainings for team members carrying out phone-based HRSN interventions.

## 1. Introduction

Interconnections between social injustices, harmful social environments, and poor health outcomes have long been recognized by many health disciplines worldwide, including public health and primary care [1,2,3,4]. In recent years, we have seen a rapid increase in the expansion of social and medical integration efforts in the United States [5,6] due to factors such as a growing emphasis on value-based care to decrease healthcare costs and improve quality [7,8,9] combined with skyrocketing health-related social needs (HRSN) as a result of the COVID-19 pandemic [10,11,12]. The National Academies of Sciences, Engineering, and Medicine (NASEM) identified 5 ways (“The 5 A’s”) [13] in which healthcare organizations can be attentive to the social contexts of their patients, including *adjusting* care plans based on patients’ social circumstances, *assisting* patients to connect with resources for HRSN, *aligning* efforts across healthcare and community-based organizations (CBOs), and *advocating* for policies that may reduce and better respond to community-level social problems. All these activities are underpinned by maintaining an *awareness* for patients’ HRSN (e.g., housing instability, food insecurity), which is frequently achieved using healthcare-administered screening tools [14,15,16].

Screening patients for HRSN has the potential for both positive and negative consequences. On the one hand, systematically screening patients for HRSN may improve healthcare quality and outcomes [17] and multiple studies indicate patients are generally supportive of such practices [18,19,20,21]. On the other hand, researchers and practitioners have raised concerns about the potential for harm in the absence of thoughtful planning and implementation of healthcare-based social interventions [22,23,24]. In particular, patients may feel judged, face discrimination, or even experience a traumatic event as a result of disclosing HRSN (e.g., losing child custody) [25,26]. Patients may also become frustrated or discouraged if they are unable to access resources for the HRSN they disclose [22,23].

The COVID-19 pandemic—which prompted an abrupt shift to telehealth [27,28]—added an additional layer of complexity to the implementation of HRSN screening. With many healthcare organizations now conducting social interventions using telehealth approaches [29,30,31], it is important to consider how to engage with patients around topics as sensitive as HRSN via remote interactions. Yet a limited number of studies to date have sought out the insights of patients who have participated in such interventions [23].

To maximize the potential for healing and minimize the potential for harm, it is vital to incorporate patients’ perspectives into the planning and implementation of healthcare-based social interventions [24]. Our study had two interrelated objectives. We sought to (a) better understand the experiences and guidance of patients who participated in a phone-based HRSN screening and referral intervention during the COVID-19 pandemic, and (b) use that information to develop recommendations for improving the patient centeredness of such interventions.

## 2. Materials and Methods

### 2.1. Qualitative Approach

We followed the Standards for Reporting Qualitative Research (SRQR) [32] and used a pragmatic paradigm to guide our approach [33]. Pragmatism is in alignment with social justice and health equity by placing an emphasis on research that is democratic, collaborative, contextual, and action-oriented [33,34,35,36,37]. Accordingly, we sought input from a wide range of stakeholders throughout the research process, took steps to ameliorate barriers to participation, and centered our analysis on areas in which healthcare organizations may have the most agency to avoid harm and cultivate healing regarding patients’ experiences with healthcare-based social interventions.

### 2.2. Context, Setting, and Intervention

We focused on the experiences and perspectives of Medicare and Medicaid beneficiaries who participated in the Accountable Health Communities (AHC) Model in Oregon. Medicare and Medicaid are government-funded health insurance programs in the U.S. serving older adults, disabled persons, and persons meeting low-income thresholds. The AHC Model is an intervention that was developed by the Centers for Medicare and Medicaid Services (CMS). It is currently being implemented in 21 states, including Oregon, to test whether systematically identifying and addressing patients’ HRSN decreases healthcare costs and utilization [38]. Community-dwelling Medicare and Medicaid beneficiaries who consent to participate in the AHC Model intervention are screened for 5 HRSN—housing instability and quality, food insecurity, transportation needs beyond medical transportation, utility needs, and interpersonal safety—using a standardized screening tool [15]. Those who screen positive for ≥1 HRSN are offered a Community Resources Summary (CRS). Those who screen positive for ≥1 HRSN plus ≥2 emergency department visits within the previous year are offered both a CRS and navigation services to facilitate connections between the beneficiary and community resources.

The current study focuses on two clinical delivery sites in Oregon: an urban emergency department (ED) affiliated with an academic medical center and a federally qualified health center (FQHC) with multiple locations across two urban and peri-urban counties. Screening and navigation services at these sites occurred over the telephone, but beneficiaries could choose to receive their CRS via text message, email, or mail. HRSN screening calls were made by health sciences students employed by the university-based department leading the implementation of the AHC Model intervention in Oregon. ED Navigators were employed by a statewide community-based nonprofit organization and FQHC navigators were employed by the FQHC.

### 2.3. Sampling Strategy

Our study included adult AHC Model intervention participants who had both qualified for and accepted navigation services. We purposefully recruited these eligible participants using a maximum variation sampling strategy to include a wide range of experiences and perspectives and identify themes that transcend a heterogeneous sample [39]. We sought variation across multiple characteristics and circumstances for which we had data, including beneficiaries’ age, sex, race, ethnicity, primary language (English or Spanish), highest level of education, and types and total number of HRSN. Additionally, our goal was to recruit a similar number of beneficiaries from the ED and FQHC.

We used the concept of “information power” to iteratively guide adequate sample size [40]. Given our pragmatic approach, broad study aims, and intention to conduct an exploratory cross-case analysis we knew a priori that a relatively large sample size for qualitative research would be needed. Throughout the data collection process, our ability to conduct purposeful sampling and rich dialogue across the majority of the interviews determined the sample size necessary to make substantive contributions to current understandings of patients’ experiences and recommendations regarding healthcare-based social interventions.

### 2.4. Ethical Issues Pertaining to Human Subjects

The institutional review board (i.e., research ethics committee) of Oregon Health & Science University approved the study (STUDY00018168). Eligible beneficiaries had already consented to participate in the AHC Model intervention, but provided additional verbal consent to participate in interviews.

### 2.5. Recruitment and Data Collection

We conducted interviews by phone and audio-recorded conversations. All interviewees received USD 25 gift cards for their time and contributions. The first author recruited eligible beneficiaries to participate, making up to three calls unless a beneficiary’s number was not working, or they requested to not be contacted again. She carried out recruitment in either English or Spanish, depending on the beneficiary’s language preference at the time of the recruitment call. Those who requested English interviews could choose to participate immediately or be called back at a time that was better for them. A bilingual and bicultural author (MF) conducted interviews in Spanish at a later time.

The first author collaborated with members of the AHC Model intervention team to develop an interview guide [41]. She also received feedback on the interview guide from a health services researcher with extensive experience creating interview guides and interviewing Medicaid beneficiaries for the Oregon Health Insurance Experiment and other projects [42]. The interview guide included questions and prompts related to participants’ health, what they remembered about the AHC Model intervention HRSN screening call, their experiences regarding follow-up activities, and information about whether and how they were able to make resource connections. The interview guide was translated into Spanish by an IRB-qualified translator. The interview guide is available as Appendix A.

### 2.6. Data Processing and Analysis

We used reflexive thematic analysis to analyze our data [43]. Reflexive thematic analysis is a flexible approach in which our analysis team could make several decisions based upon our goals and research questions. For instance, we analyzed our data inductively versus deductively, meaning that our development of codes and themes came from the data itself rather than an a priori theory or framework. We also analyzed our data semantically (i.e., exploring meaning at an explicit or manifest level) [44]. Finally, our approach was experiential in that we sought to understand participants’ own experiences, perspectives, and understandings regarding the AHC Model intervention versus a more critical approach in which multiple layers of meaning are unpacked [44].

Five authors (ASR, MF, KGA, CB, and MK) were involved in the qualitative analysis process, bringing a range of experiences and viewpoints to the creation of codes and themes. Three members of this analysis team had experience screening beneficiaries for HRSN as part of the intervention. Some of the analysts have received various types of public and non-governmental assistance for HRSN while others have not. One team member has professional working proficiency in Spanish [45,46] and two of us are bilingual and bicultural. Our analysis team met weekly from late January to early April 2022 for coding and theme development.

All interview recordings were professionally transcribed, and we analyzed the transcriptions in their original language. We created a codebook for the purpose of enhancing our understanding and reflexivity across our large team. The first author purposefully selected a sample of seven transcripts and inductively developed an initial codebook in excel. The remaining four analysts (MF, KGA, CB, and MK) used respective subsets of three of these transcripts to make their own codebooks following the same process. Next, the first author incorporated ideas from all analysts’ codebooks to make an updated version that the entire team reviewed and provided feedback on. We then uploaded the amended codebook and all transcripts to Dedoose Version 9.0.17 (Dedoose, Los Angeles, CA, USA) [47]. Each transcript was analyzed and double-coded by the first author and one of the other four analysts. We also built an excel document in which we could keep track of questions and memos to discuss as a team in our weekly analysis meetings. After completing the coding process, the first author developed preliminary themes that the full analysis team then refined, in an iterative manner, over the course of several weeks. Prior to finalizing themes, the first author checked candidate themes against the original transcripts and reviewed them with all co-authors to ensure that themes were internally coherent, distinct, and had useful clinical implications [48].

## 3. Results

From 20 August to 18 November 2021, we attempted to contact 92 beneficiaries, of which 34 (37%) participated. We determined that 34 interviews was sufficient based on the principles outlined in the concept of “Information Power” [40] described above. Participant demographics are summarized in Table 1. The sample was heterogeneous in terms of participants’ age (range: 19 to 70), highest level of education (elementary school through college), sex, race, ethnicity, and language. Every type of HRSN that the intervention screens for was represented in the sample. Interviews averaged 27 min (range: 11 to 62 min).

**Figure 1:**
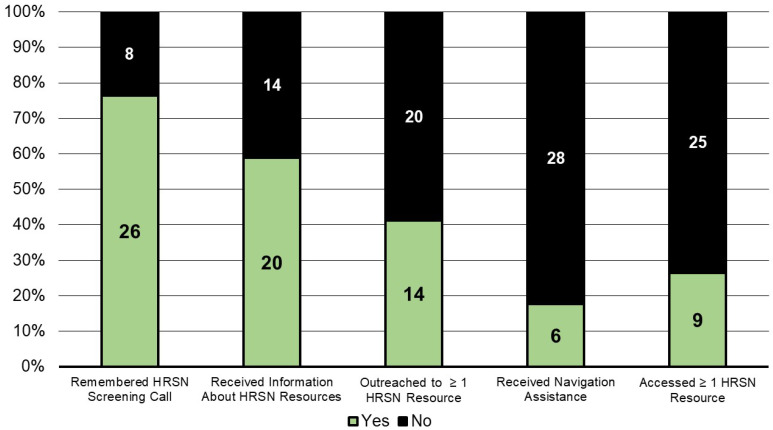
Interview participants’ self-reported AHC Model intervention outcomes (n = 34).

When asked about the AHC Model intervention, 26 participants (76%) remembered the initial HRSN screening call; 20 (59%) received information about HRSN resources, 14 (41%) attempted to connect with HRSN resources, 6 (18%) remembered receiving follow-up navigation assistance, and 9 (26%) were able to access at least 1 HRSN resource (e.g., food banks, rental assistance, bus passes) because of the intervention (Figure 1).

**Figure 2:**
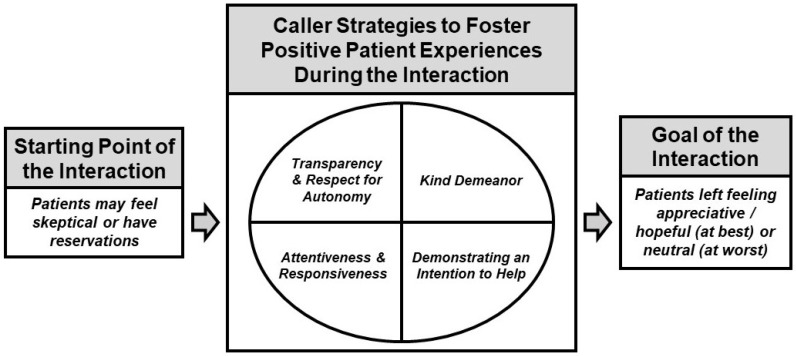
Framework for fostering positive patient experiences during phone-based HRSN screening and referral interactions.

We identified 6 themes and organized them by how they correspond to different time points of the AHC Model intervention (Figure 2). The first theme relates to the state of mind participants initially bring to the HRSN screening call. The middle 4 themes reflect dynamics and activities that occur during the HRSN screening call. The final theme relates to participants’ state of mind following the HRSN screening call. We include both the translation and the original language for all Spanish language quotes. Figure 2 shows a framework for generating positive patient experiences during the HRSN screening call based on the 6 themes. We describe each theme in detail below.

**Theme** **1.**  *Patients are likely to be initially skeptical and/or have reservations about the HRSN questions*.

Participants told us that they were (or others would be) initially somewhat skeptical due to receiving a call from an unknown number, especially with the existence of phishing scams. As one person said,

*“Some people will take that as a straight up scam, like you’re trying to track them or some shit, you know what I mean?”*

<25-year-old White male (a)

Another person shared their own reluctance when it comes to engaging with unknown callers.

*“I’m very inquisitive when it comes to that … At first [I ask], ‘Where are you calling me from? Why are you calling me?’ Not just anyone is going to be helping a person. Sometimes they just do it to grab your information.” (“Yo soy muy preguntona para eso … Al principio [yo digo], ‘¿De dónde me llamas? ¿Por qué me llamas?’ … No cualquiera va a estar ayudando a una persona. A veces nomás hacen por agarrar información.”*)

45 to 54 year-old Hispanic female

Interviewees also talked about how the sensitive nature of HRSN screening questions could make people hesitant to participate. Reasons for the questions being sensitive were embarrassment around needing help and/or concerns for whether accepting help could create problems for the person (e.g., prompting the involvement of child welfare or immigration customs enforcement). According to one person,

*“There are people out there that need help but don’t wanna be a bother … People my age and older, you don’t take handouts, that’s embarrassing.”*

45 to 54-year-old White female

Another person described a family member’s trepidation around whether or not to accept help with resources.

*“[She] didn’t want things to come down to her, like, because I’m applying for assistance then they would come and look at her, like, ‘Why aren’t you giving more money or why aren’t you doing something?’ … I think [there was a concern around something like Department of Human Services (DHS) involvement] … With my daughter’s disability there was DHS involved quite a bit in our household … Maybe it would cause her more problems to admit to some of this stuff.”*

65 to 74-year-old White female

**Theme** **2.**  *Immediate transparency and ongoing respect for autonomy are fundamental.*

Participants explained how immediate transparency regarding the purpose of the call was helpful in moving from a place of skepticism to understanding that the call was legitimate. In particular, people felt assured knowing that the call came from a healthcare organization that they were familiar with. One person said,

*“It seemed like a scam at first, but because I know [the healthcare organization] and I’ve been going there since I was like two years old, I was like, ‘No, there’s no way it’s a scam.’”*

<25-year-old White female (a)

A member of our team summarized another participant’s recommendations on the importance of transparency.

*Interviewer: “To make sure I’m getting this right, being prepared for when the person answers to give a clear description of who you are and where you’re coming from?” Interviewee: “Right. You got it. A plus.”*

65 to 74-year-old Black male

Ongoing respect for autonomy was also valued by participants. This entailed establishing with the person that they could choose not to answer any of the questions or end the conversation at any point. Additionally, it meant not pushing or hassling people for information. Examples of such sentiments included:

*“She mentioned there was going to be some personal questions and I didn’t have to answer if I didn’t want to … I appreciated that a lot.”*

<25-year-old White female (b)

*“I mean, I don’t want to be hassled if I tell them that everything is good … If I’m not in a good place, I’ll ask them. I don’t want to be pressured or hassled.”*

45 to 54-year-old Black male

**Theme** **3.**  *Showing kindness for the patient through one’s demeanor is important.*

Participants often cited callers being “nice” or “kind” as a key component of the intervention. For example,

*“It’s pretty straightforward … We all know good customer service. Be nice to people. Treat others how you’d want to be treated.”*

35 to 44-year-old Black male

Multiple aspects of a caller’s demeanor were indicative of kindness to interviewees. Listening, gentleness, and patience were some of the qualities that participants picked up on the most, and typically through a caller’s tone of voice. The following examples include two instances in which interviewees had positive interactions and one in which the interaction went poorly.

*“The tone of voice she maintained the whole time was also really helpful … Just maintaining maybe a soft, it doesn’t always have to be soft, but just like a calming [voice] … It’s very stereotypical, but it does work.”*

<25-year-old Hispanic female (a)

*Interviewer: “How did you feel during the call?” Interviewee: “Supported.” Interviewer: “Supported. Was that because of the questions they were asking you or because of the tone of their voice or both?” Interviewee: “Because of both things.”* (*Entrevistadora: “¿Cómo se sintió durante esta llamada?” Entrevistada: “Apoyada.” Entrevistadora: “Apoyada. ¿Es por las preguntas que le hicieron o por el tono de voz o ambas? Entrevistada: “Por ambas.”*)

55 to 64-year-old Hispanic female

*“She wasn’t very kind, too. Just quick and short … The tone in her voice, it seemed like she was in a big hurry … I had the feeling she didn’t have her morning coffee … There was just no life and no concern, no personal interest in what she was saying.”*

55 to 64-year-old White female

**Theme** **4.**  *Demonstrating a genuine intention to connect patients with resources matters.*

Participants said that their motivation to engage with the HRSN screening call was affected by their impression about whether the goal of the intervention was truly to connect them with resources. As one person put it,

*“As long as I think it’s gonna help me and not hurt me, I’m willing to answer questions.”*

45 to 54-year-old American Indian or Alaska Native female

Participants said that they could tell by a caller’s demeanor and tone whether they actually cared about making an effort to connect them with resources. Interviewees understood that resources are often unavailable and cannot be guaranteed, but they still needed to feel like the caller was going to do what they could to help them out.

*Interviewer: “Is it okay to ask [about social needs], even when help or resources cannot be guaranteed?” Interviewee: “It depends on the person. Look, there are times when, if they are going to help you, that’s fine! But if they are one of those people who doesn’t want to help, they will not explain it to you. Interviewer: “So, more like what are the intentions [of the person]?” Interviewee: “Yes.”* (*Entrevistadora: “¿Está bien que se hagan este tipo de preguntas, aun cuando no es posible garantizar la ayuda o los recursos?” Entrevistado: “Depende de la persona. Mira que hay veces que, si te van a ayudar, ¡está bien! Pero si son de esas personas que no quieren ayudar, no te van a explicar. Entrevistadora:* *“O sea, más bien ¿cómo son las intenciones?” Entrevistado: “Ajá.”*)

45 to 54-year-old Hispanic male

*Interviewee: “It was just very helpful … One of those things where it’s like I point out a problem [and they say] ‘Oh, there’s an issue, let’s get that taken care of.’” … Interviewer: “It sounds like you appreciated that … she had an optimistic outlook on it, but she also explained that it couldn’t be guaranteed. Like, she tried to do both of those things?” Interviewee: “Exactly. That feels more honest because you can still be optimistic without knowing that there’s a guarantee.”*

<25-year-old White male (b)

**Theme** **5.**  *The degree of attentiveness and responsiveness to patients’ circumstances and requests impacts their experience.*

Participants’ experiences with the AHC Model intervention, including the HRSN screening call, were impacted by callers’ attentiveness and responsiveness to their circumstances and requests, both at the time of the call and with follow-up activities. Regarding the call itself, several interviewees pointed out that callers need to be alert to whether patients are in a physical and emotional space where they can engage with the intervention. Further, if a caller realizes that a patient is not in a good place to participate, they should be responsive by discontinuing the intervention and trying back at another time. According to a participant with a mental health disorder that causes hallucinations,

*“If you had gotten me, say, seven months ago … it would have been significantly more difficult because I was delusional … If [a person] is so far gone that they can’t actually answer the questions, [you] just need to contact them at a later date because it’s a day-to-day thing.”*

—35 to 44-year-old male (the participant did not respond to the questions about race or ethnicity)

Regarding follow-up activities, participants were pleased when there was accordance between the requests they had made and subsequent actions. For example, one participant described the usefulness of the way in which she received information about resources,

*“I think [the process to try to connect me with resources] was very easy and thoughtful … She gave me a choice to do it online or by mail … So, when I was able to get it through the mail, like I requested, I could use it for future reference if I ever needed it.”*

55 to 64-year-old Black female

For many participants, callers’ responsiveness to their requests for accommodations seemed to make the difference between potentially connecting with resources or not. This was especially the case for assistance with completing resource eligibility paperwork. Participants said they needed support with paperwork due to language barriers, the complexity surrounding the questions asked, and fear of repercussions for making a mistake.

*Interviewee: “Oh yeah, [having someone walk me through the paperwork was helpful]. For people like me, I have a hard time with reading and things that are pretty basic … Interviewer: “Do you think that had you not had that help, you may have not applied for the bus passes, for example?” Interviewee: “Yeah, probably, because I’m just really anxious about stuff like that, especially in regards to paperwork and legal stuff, I would have been too afraid of doing it wrong.”*

<25-year-old Hispanic female (b)

Other opportunities for responsiveness related to participants’ needs around the geographic location of resources and technological accommodations. In the following examples, participants expressed frustration by the follow-up activities they experienced in those areas.

*“Well, I told him, ‘I live in [County A], so do you [have] anything in [County A]?’ But they gave me the [number for] [County B] … That’s the problem … I don’t need [County B].”*

45 to 54-year-old Asian male

*“I told him over and over again, ‘I don’t need all of the other stuff, the dot coms and all that crap.’ … [The resources were sent] as texts on my phone, but there weren’t phone numbers on any of them, and I don’t have a computer, or a laptop, or [know] how to do that.”*

55 to 64-year-old Native Hawaiian or Other Pacific Islander female

**Theme** **6.**  *Patients can be left feeling appreciative or hopeful, whether they access resources or not.*

Many participants described being left with a sense of appreciation or hope due to the HRSN screening call, whether they accessed resources or not. For example, an interviewee who was able to access food and transportation-related resources stated,

*“It’s giving so much hope and kindness … Because of COVID … because of my heart condition and health condition … I have to stay away from people, I don’t have the vaccine yet because of my heart and everything. So I’m not as social as I used to be. And some people, their lights go dim. And you guys are like the lighthouse on the beach, saying, ‘Here’s the light, I’m trying to shine it to you.’”*

45 to 54-year-old Multiracial female

Similarly, when asked about their experience being screened, a participant who did not access resources said,

*“I was happy because it made me feel like things are starting to change in society … I really felt important and like things are starting to change.”*

25 to 34-year-old Hispanic female

It is also noteworthy that a participant not remembering the HRSN screening call did not necessarily mean that they had a bad experience with the call. In fact, one interviewee pointed out that they likely had a good experience because they did not remember it. They said,

*“I don’t remember exactly, but when someone isn’t very nice to you, you would remember it … I think the person was nice. They were nice because of that, because I don’t really remember.”* (*“No recuerdo con exactitud, pero cuando alguien no es muy amable con uno, uno sí lo recuerda … Yo pienso que la persona fue amable. Fue amable por eso, [porque] no tengo mucho recuerdo.”*)

65 to 74-year-old Hispanic male

## 4. Discussion

By interviewing a diverse group of patients with HRSN who had participated in the AHC Model intervention in Oregon, our qualitative study offers several practical insights into how healthcare organizations can improve the patient-centeredness of phone-based HRSN screening and referral interventions. Positive intervention components include leading with transparency about the purpose of the call and consistently respecting patient autonomy; maintaining a kind demeanor; demonstrating a genuine intention to connect patients with resources; and being attentive and responsive to patients’ circumstances and requests. Such practices may help overcome patients’ initial hesitations, avoid harm, and leave patients with a sense of appreciate and/or hope. We used these themes to develop a framework for how to foster positive patient experiences during phone-based HRSN screening and referral interactions (Figure 2).

The prevalence of phone-based HRSN screening and referral interventions is increasing due to both the COVID-19 [11,27,29] pandemic and concerns about time constraints during clinical visits [49,50,51,52,53]. Many aspects of our themes affirm findings from several recent studies on in-person HRSN screening and referral interventions that have also placed emphasis on patient perspectives [54,55,56]. For example, robust evidence supports our theme that patients are likely to hold skepticism and reservations around HRSN screening questions, especially due to feelings of shame or fear of adverse consequences due to disclosing HRSN [54,55,56,57,58,59]. Our findings also suggest that initial skepticism may be heightened when it comes to phone-based interventions. Therefore, it is vital that healthcare personnel making the HRSN screening calls have an awareness for the sensitive nature of the questions and that policies be in place to prevent reporting when it is unwarranted (e.g., criminalizing poverty versus flagging legitimate cases of abuse or neglect) [23].

The themes that informed our positive intervention components in Figure 2 also mirror results from current studies and key principles from concepts like trauma-informed care [60] and empathic inquiry [61], particularly the importance of clearly explaining the purpose and scope of the HRSN intervention to patients and consistently respecting patient self-determination [55,56,60,61]. Moreover, our finding that both kindness and demonstrating an authentic attempt to help impact patients’ interest in participating and experiences with the intervention reflects other studies that have made similar distinctions [55,56]. For participants in our study, callers’ attentiveness and responsiveness to their situations—while significant in and of themselves—were also indicative of kindness and genuine intention to help. This fifth theme also relates to the centrality of working collaboratively with patients to understand what they need and how to support them [24,60].

Our findings advance the current literature in three key ways. First, our themes indicate that previously identified positive components of in-person HRSN screening and referral patient-provider interactions are also salient and possible to cultivate when it comes to phone-based interactions. Next, we grounded our framework for fostering positive patient experiences within a HRSN screening call interaction timeline (i.e., beginning, middle, and end) to make it more relevant for clinical practice. Finally, the “Goal of the Interaction” within our framework highlights our finding that, while callers should aspire—first and foremost—to connect patients with resources, they should also be trained to prioritize positive interactions that, at the very least, avoid harm.

Our results have implications for who healthcare organizations hire to do this work, how they are trained, and the conditions and supports they are provided with to increase the likelihood of meaningful patient engagement. Healthcare organizations may consider hiring professionals with comparable skillsets to the ones outlined in our framework (e.g., personal health navigators, community health workers). However, the fact that most participants in our study described positive experiences with the health sciences student workers that called them suggests that non-specialized professionals can also be trained to successfully carry out these interventions if needed. Regardless of who is hired, callers need sufficient time to establish trust, build rapport, and listen and respond to what patients share with them. To sustain staff capacity for empathy and avoid burnout, employers should endeavor to provide working conditions that promote staff well-being [61,62].

As is typical of other research studies on HRSN screening and referral interventions, the minority of our sample (26%) connected with resources as a result of the AHC Model intervention [63,64,65,66,67,68]. Our study may also help deepen explanations for what factors drive the amount of resource uptake following HRSN screening. For example, many of the participants in our sample were either receiving resources through other avenues or did not view their HRSN as acute enough to seek out resources. However, several of our study participants did need resources but were unable to access them due to factors such as inadequacy of resources in the community or various kinds of inaccessibility (e.g., difficulty navigating complex systems). The COVID-19 pandemic exacerbated longstanding, unjust, and avoidable social inequities in the U.S. and, despite various economic relief initiatives, the national safety net remains insufficient to adequately respond to people’s HRSN [69,70,71,72,73,74,75,76,77,78]. Therefore, it is essential that, beyond HRSN screening and referral, healthcare organizations also place more emphasis on the *alignment* and *advocacy* components of the NASEM social care integration “5 A’s” framework, especially when it comes to upending racist policies and practices [79,80,81] that perpetuate upstream population-level social disparities and downstream HRSN [24].

### Limitations

Our study had some important limitations. First, largely for ethical reasons, we only recruited people who had already consented to both participate in the AHC Model intervention and receive follow-up navigation assistance by phone. As a result, we are likely missing perspectives from those with more discomfort around HRSN screening questions and/or phone-based interventions. In addition, our participant recruitment was mostly limited to people living in urban areas, which tend to have more access to social services than rural areas [73,82]. Despite missing these perspectives, our study benefited from a maximum variation sampling approach in which we purposefully sought out the widest range of viewpoints possible to inform our results. Another limitation was that 8 participants (24%) in our sample did not remember the initial HRSN screening call. However, those individuals were still able to share their opinions and perspectives regarding phone-based HRSN interventions, in general. Finally, while some interviews provided richer dialogue than others, every interview generated pertinent data for theme development.

## 5. Conclusions

In this study, we identified six themes that impact patient experiences with phone-based HRSN screening and referral interventions. We also created a framework to assist healthcare organizations to make these interventions more patient-centered and reduce the potential for harm. Specifically, our framework may be incorporated into trainings that healthcare organizations provide to team members tasked with conducting phone-based HRSN interventions. Beyond the goal of connecting patients with needed resources, team members implementing HRSN activities should also be trained to prioritize the cultivation of a positive dynamic with patients. In light of these findings, we argue that it is critical that HRSN outreach workers receive sufficient time and support to build rapport and generate meaningful interactions with patients. Finally, beyond the work of HRSN screening and referral, it remains vital that healthcare organizations advocate for upstream policies and practices that prevent the ubiquity of downstream HRSN.

## Figures and Tables

**Table 1:** Participant demographics, HRSN, and AHC Model intervention information (n = 34).

Demographics	N (%)
Sex	
Female	20 (59%)
Male	14 (41%)
“What is your race?” (Select all that apply) *	
American Indian or Alaska Native	2 (6%)
Asian	1 (3%)
Black or African American	5 (15%)
Native Hawaiian or Other Pacific Islander	1 (3%)
White	17 (50%)
Other	7 (21%)
No Response	3 (9%)
“Are you Hispanic, Latino/a, or Spanish Origin?”	
Yes	10 (29%)
Spanish Language Interview	
Yes	5 (15%)
Age	
<25	7 (21%)
25–34	3 (9%)
35–44	2 (6%)
45–54	9 (26%)
55–64	7 (21%)
65–74	6 (18%)
“What is your highest grade or year of school you completed?”	
Never attended school or only kindergarten	0 (0%)
Grades 1–8 (Elementary)	4 (12%)
Grades 9–11 (Some High School)	4 (12%)
Grade 12 or GED (High School)	11 (32%)
College, 1–3 Years (Some College)	11 (32%)
College, 4 Years or more (College Graduate)	4 (12%)
HRSN	
Type(s) *	
Food	27 (79%)
Housing	26 (76%)
Transportation	16 (47%)
Utilities	8 (24%)
Interpersonal Safety	3 (9%)
Quantity	
1	8 (24%)
2	10 (29%)
3	12 (35%)
4	4 (12%)
5	0 (0%)
AHC Model Intervention & Interview Timing	
Clinical Delivery Site	
ED	16 (47%)
FQHC	18 (53%)
Months from AHC Model Intervention to Interview	
1	2 (6%)
2	18 (53%)
3	9 (26%)
4	2 (6%)
5	3 (9%)

* Instances in which percentages do not add up to 100.

## Data Availability

The data presented in this study are available on request from the corresponding author. The data are not publicly available due to privacy and ethical considerations.

## Appendix A. Interview Guide

Introduction

So I would like to start by talking a bit about your health, how it is at the moment, and how it might have changed recently.

- How is your health status in general? Would you say it is very good, good, relatively good, bad, or very bad?
- How would you describe your health over the last six to twelve months?

AHC Social Needs Screening Call

Back in [month], someone from our team called you on behalf of [clinical delivery site] to ask questions about resources in the community for which you might be eligible. You discussed topics like your access to food and housing.

- Do you remember that call?

If interviewee remembers the call:

- What do you remember about the questions?
- How did the interaction feel to you?
- Did the questions seem relevant to your health?
- Were you surprised to get questions about these topics from [clinical delivery site]?
- What do you think about healthcare organizations asking these types of questions, in general?
- Do you have recommendations about how to ask these questions? For example, do you have a preference for being asked these questions in person or over the phone?

If interviewee does not remember the call:

- That is totally fine. The questions were about things that may affect your health, like food, housing, and access to transportation.
- What do you think about healthcare organizations asking these types of questions, in general?
- Do you have recommendations about how to ask these questions?

Accepting Navigation for Social Needs

If interviewee remembers the call:

Our records show you accepted help to learn more about resources that may be available to you.

- Did anyone follow up with you about this? If so, what do you remember about that conversation? Probes, if needed: For example, how the conversation went, what the person asked, how you felt?
- If no one followed up, do you have thoughts about that?

If interviewee does not remember the call:

- If your healthcare provider offered to help you identify resources for things like food and housing, do you think you would accept their support? Why or why not?

Accessing Resources for Social Needs

If interviewee remembers the call:

- Were you able to access any resources through the person who followed up with you or through your own outreach?
- If so, please share a bit about the resources you received. Did you receive the resource information through the mail or electronically?
- If not, do you have thoughts about that?
- Are you still facing any of the same problems you were experiencing before?
- Are you okay with healthcare organizations asking about these types of things, even if they are not able to resolve the problem?
- Do you have any recommendations for how to make community resource referrals smoother for patients?

If interviewee does not remember the call:

- Have you tried to access community resources like these before? Why or why not?
- Are you okay with healthcare organizations asking about these types of things, even if they are not able to resolve the problem?
- Do you have any recommendations for how to make community resource referrals smoother for patients?

Conclusions

“Thank you for your time and sharing your thoughts. It will hopefully help us improve the care we provide to patients! Do you have any final comments to share?”
